# Skin-Sparing Mastectomy with Immediate Breast and Nipple Reconstruction: A New Technique of Nipple Reconstruction

**DOI:** 10.1155/2013/406375

**Published:** 2013-01-02

**Authors:** Raffaele Serra, Anna Maria Miglietta, Sergio Abonante, Vincent Giordano, Gianluca Buffone, Stefano de Franciscis

**Affiliations:** ^1^Department of Medical and Surgical Science, University Magna Graecia of Catanzaro, Viale Europa, 88100 Catanzaro, Italy; ^2^Breast Unit, Annunziata Hospital, 87100 Cosenza, Italy; ^3^Plastic Surgery Unit, Annunziata Hospital, 87100 Cosenza, Italy

## Abstract

*Background*. Most women with breast cancer today can be managed with breast conservation; however, some women still require mastectomy for treatment of their disease. Skin-sparing mastectomy (SSM) with immediate reconstruction has emerged as a favorable option for many of these patients. The authors combined the SSM technique with the preservation of a small part of the areola with immediate nipple together with with breast reconstruction. *Methods*. In an 8-year-period 155 female patients (age: 20–52 years old; mean age: 37.5 years) with extensive ductal intraepithelial neoplasia (DIN) or invasive breast cancer were treated with areola skin sparing mastectomy with immediate nipple and breast reconstruction. Patients were followed up prospectively by the breast surgeon, the plastic surgeon, and the oncologist for complications and recurrence. *Results*. After treatment, only 2 cases (1.29%) had a local recurrence. 8 out of 155 (5.5%) patients developed early complications (infections, seroma, haematoma), and 5 out of 155 patients (3.22%) developed delayed complications (implant rotation, aestethic deterioration) in the post operative time period. The final aesthetic outcome was judged as positive in 150 out of 155 patients (96.78%). *Conclusion*. In our experience, immediate nipple reconstruction after skin-sparing mastectomy is a technically feasible procedure which can give excellent cosmetic results.

## 1. Introduction

Mastectomy represents the treatment of choice for approximately one-third of women with breast cancer due to aggressive, extensive, or multicentric tumour growth, contraindications for radiotherapy, or following the patient's wish. 

To most of these cases, immediate breast reconstruction (IBR) can be offered to overcome the psychological burden caused by the disfigurement resulting from the loss of the breast [1]. 

Skin-sparing mastectomy (SSM) can be followed by immediate breast reconstruction (IBR) using autologous tissue and/or prosthetic implants, and this approach has been advocated as an effective treatment option for patients with early-stage breast cancer which is not amenable to breast-conserving therapy [2–4].

The presence of the nipples seems fundamental to marking the identity of the breast. Based on the psychological impact of nipple-areola complex (NAC) removal in classical mastectomy techniques, several authors have evaluated the risk of nipple areola involvement and investigated the possibility of nipple areola preservation, but the risk of cancer recurrence in the breast tissue preserved beneath the NAC for the blood supply is considered a major reason to avoid NAC conservation during the mastectomy [5].

The authors combined the SSM technique with the preservation of a small part of the areola with immediate nipple and breast reconstruction.

The most interesting part of the method used at the author's institution is precisely the particular new technique of nipple reconstruction that has not been previously described.

The authors reports their experience carried out in the last eight years in order to evaluate the oncological safety, postoperative morbidity and patients' satisfaction with this technique.

## 2. Material and Methods

In an 8-year-period (2003–2010), 155 female patients (age: 20–52 years old; mean age: 37.5 years) with extensive ductal intraepithelial neoplasia (DIN) or invasive breast cancer (T1 and T2 tumors and an unfavorable breast to tumor ratio) were treated with areola skin-sparing mastectomy with immediate nipple and breast reconstruction.

Demographics and patient characteristics are shown in Table 1.

All patients were evaluated preoperatively by a multidisciplinary team, including the breast surgeon and the plastic surgeon, to define appropriate surgical procedure and to explain to the patient the advantages and possible disadvantages of the selected option.

Informed written consent was obtained and in this study the principles outlined in the Declaration of Helsinki have been followed.

**Figure 1:**
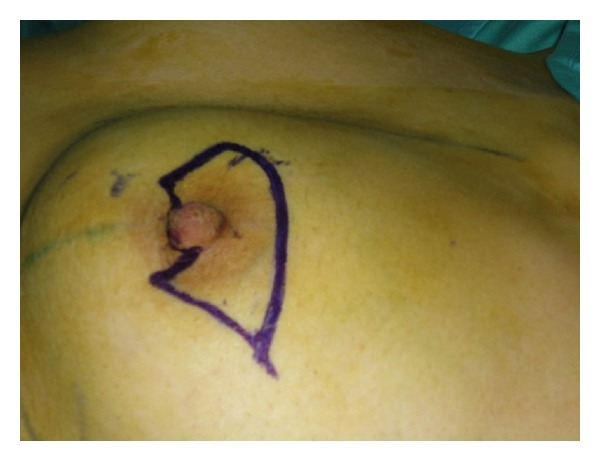
Incision line preserving part of the areola.

**Figure 2:**
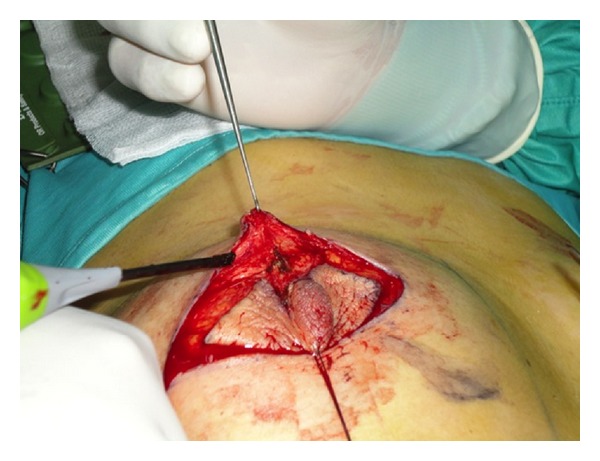
Surgical incision.

The standard Stewart elliptical skin incision was performed around nipple/areola complex while preserving, as an originality of the tecnique, a small part of areola which will be used later for the reconstruction of the nipple (Figures 1 and 2). For oncological safety, the part of the areola to preserve is selected in order to be sufficiently distant from the tumor, and preferably on the opposite pole to it, but also to allow a symmetrical positioning with respect to the contralateral nipple.

Subsequent surgical procedures consisted of removal of mammary tissue and nipple-areola complex (NAC) with the preservation of the remaining breast skin envelope and the inframammary fold. 

Once SSM has been completed, breast reconstruction commences with an incision along the lateral border of the pectoralis major muscle, and a submuscular pocket is created deep to both the pectoral and serratus muscles. To prevent cranial displacement of the implants and to allow for a more natural projection of the lower part of the reconstructed breast, the origin of the major pectoral muscle was detached from the lower costal arch and the caudal part of the sternum. 

In this pocket, an appropriate size of the textured prosthesis (textured profile (anatomical) saline implants; mentor; california-USA) is placed.

The Closure of the pocket was achieved by using the pectoralis major muscle as coverage in its upper two-thirds and using the mastectomy skin flap as coverage in its lower third in order to avoid upper-pole hyperexpansion by virtue of decreased resistance to the forces of expansion in this area and a concomitant lower-pole elevation, leaving in this way the newly mammary fold by the curvature of the breast prosthesis. 

**Figure 3:**
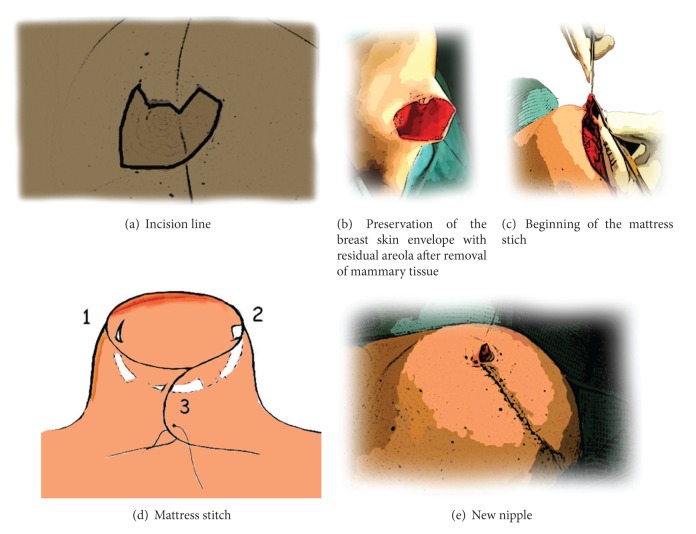
Scheme of the technique.

Before suturing the skin reconstruction of the nipple begins, an absorbable suture is positioned at the base of the truncated cone formed by the residual areola, then a mattress stitch iss put between the new nipple edges. An intradermal continuous suture on the joining edges of the new nipple is performed, and at the end of the procedure, skin suturing is performed (Figure 3).

In our case series only unilateral reconstruction was performed.

All patients were followed during the postoperative period by the breast surgeon, the plastic surgeon and the oncologist. Followup was programmed every week for the first month, and monthly for the following 6 months, then every 6 months. The average followup was 4.5 years.

Patient satisfaction was estimated using a questionnaire (Table 3) administered at 1 year from the procedure.

The score was then totaled for a maximum of 10 points per patient as follows: “Excellent” (9-10 points), “Good” (7–8.5 points), “Acceptable” (5–6.5 points), and “Poor” (≤4.5 points).

Objective data on the size, projection, and the symmetry with respect to the contralateral nipple were also collected. 

The height and diameter of the reconstructed nipple were measured directly after the procedure and during follow-up controls.

After surgical procedure, 87 patients (56.12%) underwent adjuvant chemotherapy with FAC chemotherapy (5-fluorouracil, doxorubicin, cyclophosphamide) given 3 times weekly for 6 cycles, while 68 patients (49.15%) underwent long-term tamoxifen therapy for 5 years.

## 3. Results

After treatment, only 2 cases (1.29%) had a local recurrence.

Among the 155 patients who underwent SSM with immediate breast and nipple reconstruction, 8 (5.5%) patients developed early and 5 patients (3.22%) delayed complications in the postoperative time period; the complications have been collected and are reported in Table 2.

Given the thin nature of the residual areola tissue used for nipple reconstruction, there were, however, no instances of nipple necrosis as might be expected. 

There were also no evidence of capsular contractures in the followup period.

The average diameter of the reconstructed nipple in the immediate postoperative period was 11.8 ± 1.1 mm compared with 12.1 ± 0.9 mm for the opposite nipple. After 1 year, the average diameter was 13.2 ± 1.3 mm.

The average projection of the nipple in the immediate postoperative period was 6.15 ± 0.6 mm, compared with 5.12 ± 1.35 mm for the opposite side. After one year the average projection of the new nipple was 4.16 ± 0.7 mm (−15.86%).

Symmetrization was achieved satisfactorily during first procedure for almost all patients. Only in two cases (1.29%), the symmetrization of the nipple compared with the contralateral nipple has not been satisfactory (due to the location of the tumor), and it was necessary to proceed with further intervention of repositioning of the nipple under local anesthesia after six months from the first procedure.

All patients completed aesthetic questionnaire.

**Figure 4:**
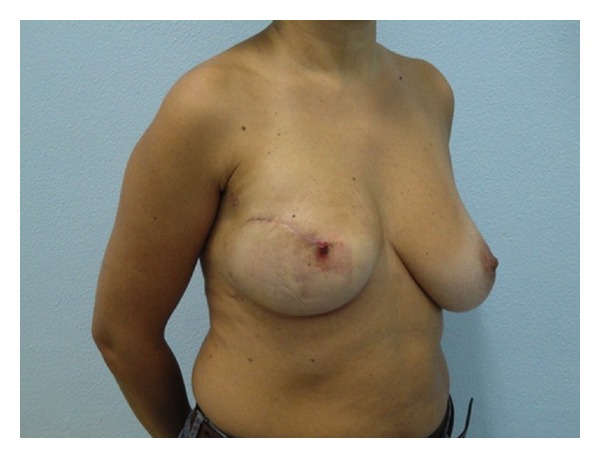
Outcome Example after 1 year.

The final aesthetic outcome was judged, according to questionnaire filled by all patients, as excellent in 98 patients (63.22%), good in 43 patients (27.74%), acceptable in 9 patients (5.80%), and poor in 5 patients (3.22%). An example of positive result after 1 year is shown in Figure 4.

## 4. Discussion

Indications for mastectomy (with/without reconstruction) included tumor size >5 cm, central sector tumor unsuitable for breast conserving surgery (BCS), multiple tumor foci, relatively large tumor with respect to breast size, extensive high-grade *in situ* carcinoma, inflammatory cancer, history of prior cancer in the breast, where radiotherapy is contraindicated, and patient preference. In the absence of all these factors, BCS followed by adjuvant radiotherapy was the treatment of choice.

Historically, malignant involvement of the nipple-areola complex (NAC) in mastectomy specimens has been cited to be 5.6%–58%, thus leading many to suggest that NAC preservation is not a reasonable option [6, 7].

Concerned about local-regional recurrence from occult neoplastic involvement of the NAC, Petit and collegues performed the same nipple-sparing mastectomy and added intraoperative or postoperative brachytherapy to the preserved NAC. This resulted in equivalent oncologic results but higher rates of nipple necrosis and resection [8].

An alternative to NAC-sparing mastectomy is to remove the nipple, but preserve the areola (areola-sparing mastectomy), followed, in a second step, by nipple reconstruction using one of the conventional local flap techniques. Results are controversial [9]. 

In our personal experience, we preferred saline implants for breast reconstruction as they have a lower rate of revision surgery than silicone gel, and the overall rate of capsular contracture is lower for saline than silicone [10]. Further, the scar is shorter, as saline implants can be filled after they are placed, allowing a smaller incision, and there is no need for MRI, as silent rupture is not a concern.

The objectives of nipple reconstruction aim to create a nipple which is symmetrical to the contralateral nipple precisely in terms of shape, size, position, and projection in order to achieve patient satisfaction.

Numerous tecniques have been described for nipple reconstruction following mastectomy (e.g., local adipocutaneous flap, distant tissue flaps, cartilage grafts, local flaps as bell, skate flaps, star flaps, bilobed flaps, trilobed flpas, and C-V flap technique) but no one is entirely satisfactory. In fact, the most common problem following nipple reconstruction is that projection of the new nipple tends to shrink with time, and reported figures for percentage projection loss vary significantly [11, 12].

In our casuistry, we reported an increase of 12.1% in the diameter of the reconstructed nipple and a projection loss of 15.86% after 1 year.

In our experience the low rate of recurrences (1.29%) and complications (5.5% immediate and 3.22% late) with the positive aesthetic outcome of the procedures (96.76%) allows us to state that immediate nipple reconstruction after SSM is a technically feasible procedure which can give excellent cosmetic results.

Moreover, a recent paper showed that breast-conserving therapy nowadays can be offered as standard care with long-term results, in terms of survival, comparable to that of standard procedures [13].

In our experience, our unique stage surgery saves time in the surgical theatre and reduces the number of hospitalizations by giving to the patients the chance to complete the reconstruction in one stage without delaying the breast reconstruction and achieves, at the same time, excellent cosmetic results. 

## Figures and Tables

**Table 1:** Characteristics of patients.

Number of patients	155
Age of patients	20–52 (range), 37.5 (mean)
Followup (months)	12–96 (range), 47 (mean)
Tumor size	
Extensive DIN	23
T1	36
T2	96
Histology	
Ductal	137
Lobular	18
Nodal status	
N0	151
N1	4

**Table 2:** Complications.

Early	
Infections	1 (0.64%)
Seroma	3 (1.93%)
Haematoma	4 (2.58%)

Delayed	

Implant rotation	2 (1.29%)
Aesthetic deterioration	3 (1.93%)

**Table 3:** Satisfaction Questionnaire.

Items	Very dissatisfied	Somewhat dissatisfied	Somewhat satisfied	Very satisfied
(A) How satisfied or dissatisfied have you been in the last 6 months with				
(1) the Shape of the breast	0.5	1	1.5	2
(2) the Scars of the breast	0.5	1	1.5	2
(3) the Sensibility of the breast	0.5	1	1.5	2
(4) the Appearence of the new nipple	0.5	1	1.5	2
(B) the overall surgical procedure fulfilled your expectations?				
	0.5	1	1.5	2

## References

[1] Wirth R, Banic A (2010). Aesthetic outcome and oncological safety of nipple-areola complex replantation after mastectomy and immediate breast reconstruction. *Journal of Plastic, Reconstructive & Aesthetic Surgery*.

[2] Cunnick GH, Mokbel K (2004). Skin-sparing mastectomy. *American Journal of Surgery*.

[3] Singletary SE, Robb GL (2003). Oncologic safety of skin-sparing mastectomy. *Annals of Surgical Oncology*.

[4] Simmons RM, Adamovich TL (2003). Skin-sparing mastectomy. *Surgical Clinics of North America*.

[5] Schecter AK, Freeman MB, Giri D, Sabo E, Weinzweig J (2006). Applicability of the nipple-areola complex-sparing mastectomy: a prediction model using mammography to estimate risk of nipple-areola complex involvement in breast cancer patients. *Annals of Plastic Surgery*.

[6] Cense HA, Rutgers EJT, Lopes Cardozo M, Van Lanschot JJB (2001). Nipple-sparing mastectomy in breast cancer: a viable option?. *European Journal of Surgical Oncology*.

[7] Simmons RM, Brennan M, Christos P, King V, Osborne M (2002). Analysis of nipple/areolar involvement with mastectomy: can the areola be preserved?. *Annals of Surgical Oncology*.

[8] Petit JY, Veronesi U, Rey P (2009). Nipple-sparing mastectomy: risk of nipple-areolar recurrences in a series of 579 cases. *Breast Cancer Research and Treatment*.

[9] Patani N, Devalia H, Anderson A, Mokbel K (2008). Oncological safety and patient satisfaction with skin-sparing mastectomy and immediate breast reconstruction. *Surgical Oncology*.

[10] Benediktsson K, Perbeck L (2006). Capsular contracture around saline-filled and textured subcutaneously-placed implants in irradiated and non-irradiated breast cancer patients: five years of monitoring of a prospective trial. *Journal of Plastic, Reconstructive & Aesthetic Surgery*.

[11] Jamnadas-Khoda B, Thomas R, Heppell S (2011). The “cigar roll” flap for nipple areola complex reconstruction: a novel technique. *Journal of Plastic, Reconstructive & Aesthetic Surgery*.

[12] Jones AP, Erdmann M (2012). Projection and patient satisfaction using the “Hamburger” nipple reconstruction technique. *Journal of Plastic, Reconstructive & Aesthetic Surgery*.

[13] Litière S, Werutsky G, Fentiman IS (2012). Breast conserving therapy versus mastectomy for stage I-II breast cancer: 20 year follow-up of the EORTC 10801 phase 3 randomised trial. *The Lancet Oncology*.

